# Peripherical Blood hsa-miR-335-5p Quantification as a Prognostic, but Not Diagnostic, Marker of Gastric Cancer

**DOI:** 10.3390/diagnostics14151614

**Published:** 2024-07-26

**Authors:** Lizbeth Ramírez-Vidal, Jared Becerril-Rico, Alberto Monroy-Mora, Jose Manuel Tinajero-Rodríguez, Federico Centeno-Cruz, Luis F. Oñate-Ocaña, Elizabeth Ortiz-Sánchez

**Affiliations:** 1 Posgrado de Ciencias Biomédicas, Facultad de Medicina, Universidad Nacional Autónoma de México, Circuito Exterior s/n Ciudad Universitaria, Coyoacán, Mexico City 04510, Mexico; lisbetrv@gmail.com; 2 Programa de Maestría en Ciencias Biológicas, Universidad Nacional Autónoma de México, Circuito Exterior s/n Ciudad Universitaria, Coyoacán, Mexico City 04510, Mexico; jared.becerril.rico@gmail.com (J.B.-R.); albertomonm30@gmail.com (A.M.-M.); 3 Facultad de Ciencias Químico Biológicas, Universidad Autónoma de Guerrero, Chilpancingo 39090, Mexico; jtinajeror@outlook.es; 4 Laboratorio de Inmunogenómica y Enfermedades Metabólicas, Instituto Nacional de Medicina Genómica, Mexico City 14610, Mexico; fcenteno@inmegen.gob.mx; 5 Subdirección de Investigación Clínica, Instituto Nacional de Cancerología, Av. San Fernando 22, Colonia Sección XVI, Tlalpan, Mexico City 14080, Mexico; lfonate@gmail.com; 6 Subdirección de Investigación Básica, Instituto Nacional de Cancerología 5 Av. San Fernando 22, Colonia Sección XVI, Tlalpan, Mexico City 14080, Mexico

**Keywords:** gastric cancer, miRNA, hsa-miR-335-5p, GC molecular markers, GC prognosis

## Abstract

Gastric cancer (GC) is a leading cause of death, and this pathology often receives a diagnosis in an advanced stage. The development of a less invasive and cost-effective test for detection is essential for decreasing the mortality rate and increasing the life expectancy of GC patients. We evaluated the potential targeting of CD54/ICAM1, a marker of gastric cancer stem cells, with miRNAs to detect GC in blood samples. The analyses included 79 blood samples, 38 from GC patients and 41 from healthy donors, who attended INCan, México City. The total RNA was obtained from the blood plasma, and RT-PCR and qPCR were performed to obtain the relative expression of each miRNA. Hsa-miR-335-5p was detected in the plasma of GC patients and healthy donors at the same levels. The ROC curve analyses indicated that this miRNA was not a candidate for the molecular diagnosis of GC. We did not observe a correlation between the expression of hsa-miR-335-5p and clinical variables; however, the Kaplan–Meier analyses indicated that, in patients who survived more than 12 months, a lower expression of hsa-miR-335-5p was correlated with a better prognosis. It would be convenient to evaluate a larger panel of miRNAs, including miRNAs expressed in a limited number of cell types or with a low number targets, to obtain more specific candidates for developing a robust test for the diagnosis/prognosis of GC.

## 1. Introduction

Gastric cancer is one of the leading causes of cancer deaths worldwide; it represents the fourth most common cancer-associated cause of death, with a median survival rate of less than 12 months for the advanced stages (clinical stage IV) [1]. In 2020, 1,089,103 new cases and 768,793 new deaths were reported [1,2]. GC is asymptomatic in the early stages of the disease, resulting in a late diagnosis in the advanced stages when the prognosis is poor, with a 5-year survival rate of about 20% [3].

Some of the problems to be overcome include the development of a noninvasive method of diagnosis/prognosis of GC and the need to involve qualified experts in the proper diagnosis and treatment of this disease. The conventional diagnostic procedures are invasive, as they require an endoscopy and several tissue biopsies, and furthermore, the treatment protocol requires a highly qualified pathologist [4]. Although biomarkers such as PD-L1, Her-2, and MSI (microsatellite instability) have been identified, the diagnostic procedure also requires a qualified pathologist [4,5] and invasive tools. Early detection during the resectable stage increases the life expectancy to 5 years [6,7] and could also help to decrease the mortality rate. However, the protocols for early detection recommend radiographic screening in people over 40 years, with or without risk factors [8]. Such strategies represent a very large expense for health systems and have been applied only in a few East Asian countries.

It is necessary to implement tools to develop a simple, noninvasive, and cost-effective diagnostic system, not only for patients in the initial stages of the disease, but also for the general population. One suggested solution is the use of liquid biopsies, since when tumor cells grow, they release nucleic acids such as microRNAs (miRNAs), long noncoding RNAs (lncRNA), circular RNA (circRNA), and circulating tumor DNA (ctDNA) into the blood [9]. MiRNAs can be secreted or released into body fluids [10], including blood, and can be transported to target cells in vesicles [10,11]. These molecules have great potential as biomarkers [12,13,14] for use in a noninvasive method to determine a diagnosis and/or prognosis for several diseases, including cancer.

MiRNAs are small ncRNAs with an average length of 22 nucleotides [15]. In general, miRNAs interact with the 3′ untranslated region of a target mRNA to induce its degradation or translational repression [10]. It has been reported that miR-21, miR-93, miR-106a, miR-106b, miR-223, and miR-100 are highly expressed in the serum of GC patients and, except for miR-93 and miR-106a, their expression is correlated with the TNM stage [12,16]. Furthermore, the analysis of seven GC cell lines showed that thirty-eight miRNAs were upregulated and four were downregulated [17]. Among the miRNAs reported to have an importance in GC, some function as oncomiRs while others function as tumor suppressors [18]. Finally, miRNAs are important for cancer development and metastasis [14,19]; thus, they have great potential for use in a diagnostic/prognostic noninvasive test.

A previous study conducted by our group showed the presence of a cell subpopulation with gastric cancer stem cell (GCSC) properties in the biopsies of 127 patients. These cells specifically had the phenotype CD24+CD44+EpCAM+CD54+, and their presence was correlated with a poor prognosis. In healthy donors, these GCSCs were not present; instead, we detected a cell population with the CD24+CD44+EpCAM+CD54- phenotype [20]. These data suggest that the cellular marker CD54/ICAM1 could be important for cancer development, progression, and metastasis. Considering that cells expressing CD54/ICAM1 are differentially expressed in GC patients and given the relevance of these cells in the migration, we quantified the expression of four miRNAs potentially targeting CD54 in this study. We measured the expression of hsa-miR-3186-3p, hsa-miR-3975, hsa-miR-1256, and hsa-miR-335-5p in the blood plasma of GC patients and individuals without GC to determine if these miRNAs could be used as diagnostic molecules to detect the presence of GC in the early stages of the disease. Specifically, hsa-miRNA-335-5p has been reported as a potential regulator of CD54 in different types of cancer [17,21,22].

From the miRNAs, we obtained a low AUC in the ROC curve for the evaluation of the diagnostic test, indicating that miR-335-5p is not an ideal candidate for the diagnosis of GC in blood plasma samples. However, in patients who survived more than 12 months, a lower expression of hsa-miR-335-5p was correlated with a better prognosis. These data indicated that hsa-miR-335-5p could be used as a biomarker to determine the prognosis of GC patients, but not to make a diagnosis.

## 2. Materials and Methods

### 2.1. Defining miRNAs for CD54/ICAM1

We searched the literature for miRNAs that interact with or regulate CD54/ICAM1. In parallel, we searched in GeneCards and the miRbase database (https://mirbase.org/) (accessed on 9 January 2024) for potential miRNAs that interact with the 3′ UTR of CD54 (Table 1).

### 2.2. Patients and Samples

In this study, 42 plasma samples were collected from patients with GC (male/female ratio of 44.7/55.3); however, only 38/42 samples were included (in samples discarded, no amplification was obtained for the control miRNA). In addition, 41 samples from individuals without cancer were included. The blood samples were collected after obtaining informed consent from patients with GC who visited the Instituto Nacional de Cancerología (INCan) in Mexico City from 2021 to 2024. All the patients were diagnosed, and their stage was determined according to the TNM system classification for GC. Their clinicopathologic characteristics, including their age, gender, clinical TNM stage, Borrmann’s classification, Lauren classification, and differentiation grade, were registered. These clinical characteristics are presented in Table 2. Patients who received any antineoplastic treatments were excluded from the study.

### 2.3. Total RNA Isolation and Reverse Transcription

Five-milliliter blood samples were collected in EDTA tubes (BD, East Rutherford, N J, USA cat. no. 367525). The blood plasma was obtained after a 10 min centrifugation at 1900× *g*, followed by a second centrifugation of the supernatant for 5 min at 16,000× *g*. The plasma samples were frozen and maintained at −70 °C until processed. The total RNA was extracted from 200 mL of plasma with an miRNeasy serum/plasma kit (Qiagen, cat. No. 217184) according to the manufacturer’s protocol. The RNA samples were eluted in nuclease-free water and the RNA purity was determined using A260/A280 ratios.

The reverse transcription of hsa-miR-335-5p (PN: 4427975, assay: 000546), hsa-miR-3186-3p (PN: 4440886, assay: 243084), hsa-miR-3975 (PN: 4440886, assay: 464294), hsa-miR-1256 (PN: 4427975, assay: 002850), and hsa-miR-16-5p as a control (PN: 4427975, assay: 000391) was performed with 50 nM of a specific stem-loop RT primer (from Applied Biosystems) and 10 ng of total RNA. These four circulating human miRNAs and one control (hsa-miR-16-5p) [27,28] were reverse transcribed with high-capacity RNA to cDNA using a reverse transcription kit (Applied Biosystems, Waltham, MA, USA, cat. no. 4387406) according to the manufacturer’s protocol. All the samples were run in triplicate with a final volume of 20 μL for the reaction.

### 2.4. Quantitative PCR

All the qPCR reactions were performed in triplicate with a 2X TaqMan Universal PCR Master Mix kit (Applied Biosystems, cat. No. 4304437) using TaqMan MicroRNA assay primers for hsa-mi-335-5p or hsa-mi-16-5p (Thermo Fisher, Waltham, MA, USA, cat. no. 4427975 assay: 000546 and assay: 000391, respectively) with the following conditions: denaturalization at 95 °C for 10 min, followed by forty cycles at 95 °C for 15 sec and 60 °C for 60 sec in a CFX Opus 96 Real-Time PCR System (Bio Rad, Hercules, CA, USA) with CFX Maestro software V5.2. The expression levels of the four miRNAs were normalized to hsa-miR-16-5p [13,29].

The relative miRNA expression was calculated according to the 2^−ΔΔCT^ method, as described by Livak and Schmittgen [30,31], to determine the relative changes in the expression in GC patients with respect to the healthy donors.

### 2.5. Statistical Analysis

The statistical analyses were performed using SPSS v2.6 (IBM Corp., Armonk, NY, USA) and GraphPad Prisma 5.0 (GraphPad Software Inc., San Diego, CA, USA). A non-parametric Mann–Whitney U test was used to compare the miRNA levels between the different patient groups, and all the data are presented as the mean and standard deviation to compare the relative quantities of miRNA expression. The data were analyzed with a chi-square test or Fisher’s test (as appropriate) to determine the association between the expression of hsa-miR-335-5p and the clinical variables. All the subjects were included in the associative statistical analysis; however, only the subjects with a follow up time > 4 months were included in the survival analysis to avoid bias in the results from unobservable long-term outcomes in the subjects with a follow up < 4 months.

A receiver operating characteristic (ROC) curve analysis was calculated from the 2^−ΔΔCT^ value for each miRNA using the SPSS v2.6 software (IBM Corp.) to evaluate the capability of the tested miRNAs to discriminate between samples from healthy donors and patients with GC. The AUC was calculated to evaluate the performance of the diagnostic test regarding its specificity and sensitivity.

## 3. Results

### 3.1. MiRNAs That Potentially Regulate ICAM1 in Cancer

To investigate whether the miRNAs that regulate CD54/ICAM1 could be used as molecules for the diagnosis/prognosis of GC, we searched the literature to identify miRNAs that regulate ICAM1 during carcinogenesis. In parallel, we searched the miRbase (https://mirbase.org) for potential regulators of ICAM1. These results are summarized in Table 1. We measured the expression level of the four miRNAs with the highest scores (hsa-miR-335-5p, hsa-miR-3186-3p, hsa-miR-3975, and hsa-miR-1256) in the blood of healthy donors and GC patients to evaluate if they could be used in a diagnostic test.

### 3.2. Clinical Characteristics of the Patients

A total of 38 patients with GC (male/female ratio of 44.7/55.3) with a mean age of 55.66 years old (±13.34) participated in this study. The patient cohort had the following TNM stages: stage I (7.9%), stage IIA (10.5%), stage IIIA (18.4%), stage IIIB (26.3%), stage IIIC (2.6%), and stage IV (34.2%). According to their TNM stage, most of the patients had advanced or metastatic GC (47.3%), 47.3% had locally advanced GC, and 18.4% had early GC (Table 2). The histological subtype was uniformly distributed; 47.4% of the patients had intestinal GC, 47.4% had diffuse GC, and 2.6% had a mixed GC subtype. The mean follow up was 11.5 months (±15.76 months). The healthy donors (*n* = 41) had a mean age of 34.4 years old (±15.13).

### 3.3. Diagnostic/Prognostic Value of hsa-miR-335-5p

**Figure 1:**
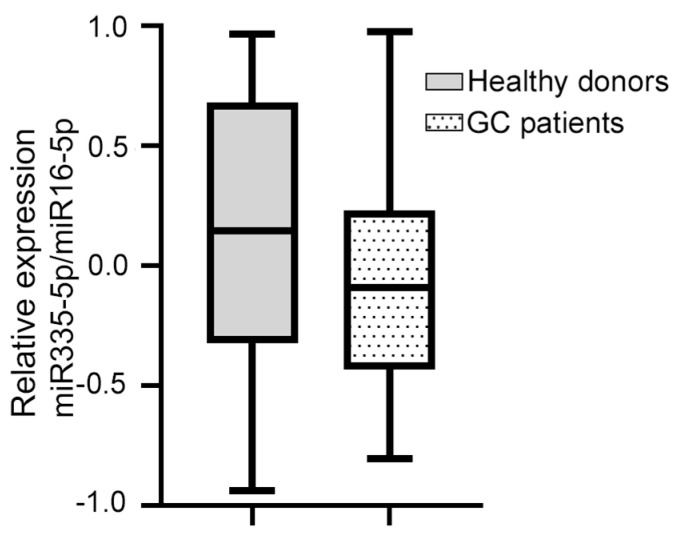
The relative expression of miR-335-5p/miR-16 in the blood plasma of GC patients and healthy donors did not change. The dotted box shows that the median relative expression of hsa-miR-335-5p/hsa-miR-16-5p in 38 GC patients was −0.092 (−0.432 +0.230); the gray box shows that the median relative expression of hsa-miR-335-5p/hsa-miR-16-5p in 41 healthy volunteers was 0.135 (−0.339, +0.684). The Mann–Whitney U test was used.

**Figure 2:**
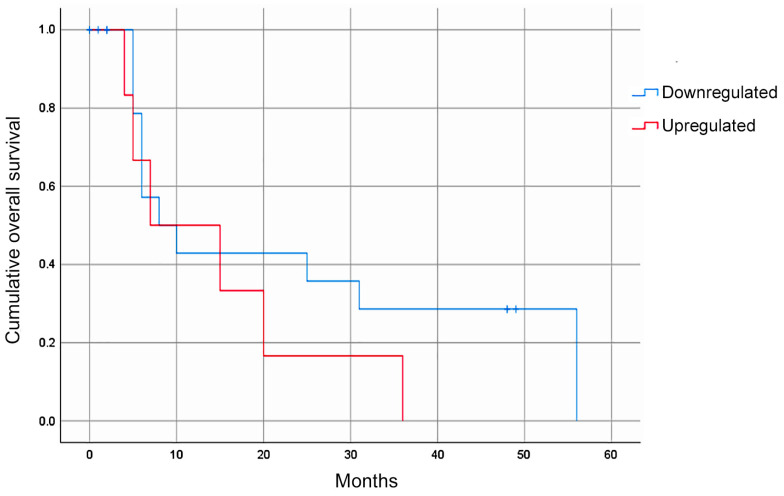
The overall survival rate of GC patients according to their hsa-miR-335-5p expression. The Kaplan–Meier gross analyses did not show an association between the hsa-miR-335-5p expression level and the overall survival (*p* = 0.271). After adjusting based on survival beyond 12 months, a decreased expression of hsa-miR-335-5p was associated with a good prognosis (*p* = 0.031).

Of the four miRNAs evaluated, only hsa-miR-335-5p was expressed in the blood plasma of the patients with GC. The median relative expression of hsa-miR-335-5p/hsa-miR-16-5p was −0.092 (–0.432 +0.230, Figure 1). The statistical analysis did not show a significant association between the relative expression of hsa-miR-335-5p and any of the clinical variables. Furthermore, the Kaplan–Meier curves for the gross analysis showed no significant differences in the survival rates between the groups with a high vs. low expression of hsa-miR-335-5p (*p* = 0.271, Figure 2). However, after adjusting the survival based on a window of time greater than 12 months, the patients with a decreased expression of hsa-miR-335-5p had a better prognosis, showing a higher overall survival after 12 months (*p* = 0.031). Therefore, the relative expression of hsa-miR-335-5p in the blood plasma is associated with a good long-term prognosis in GC patients.

We investigated whether hsa-miR-335-5p was expressed in healthy donors. The median relative expression of hsa-miR-335-5p in 41 healthy volunteers (41.5% men and 58.5% women) was 0.135 (−0.339, +0.684). On the other hand, the median relative expression of hsa-miR-335-5p in GC patients was −0.092 (−0.432, +0.230, Figure 1). We found that there was no significant difference in the relative expression of hsa-miR-335-5p between healthy volunteers and GC patients (*p* = 0.081).

**Figure 3:**
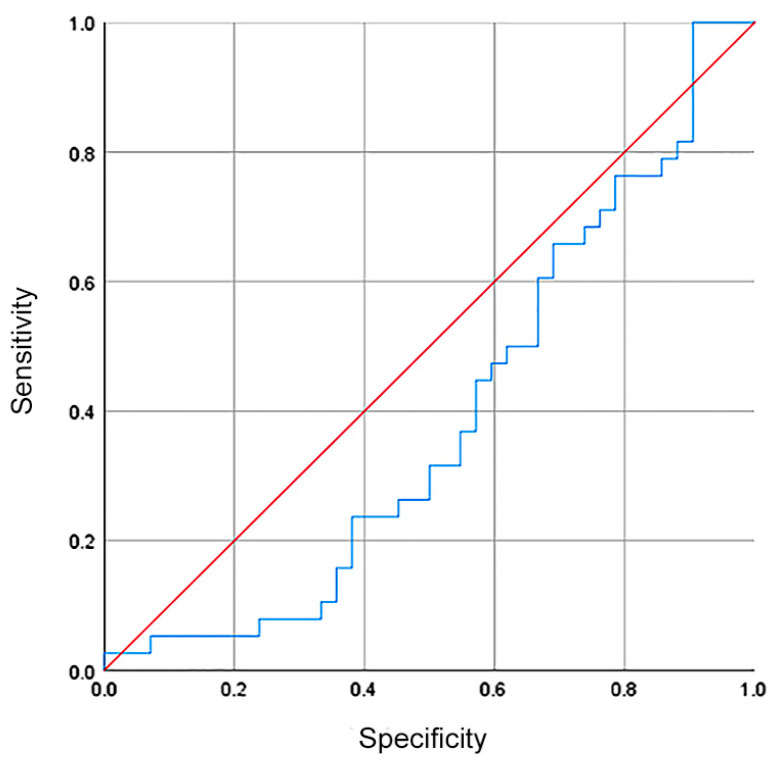
The relative expression of hsa-miR335-5p had no diagnostic value for patients with GC. The ROC curve showed a sensibility, specificity, and AUC of 37%, 44%, and 0.39 (blue line in the plot).

Although there was no difference in the hsa-miR-335-5p expression between healthy volunteers and GC patients, we investigated whether hsa-miR-335-5p could be used as a biomarker with diagnostic/screening potential. The ROC curve showed a sensibility, specificity, and AUC of 37%, 44%, and 0.39, respectively (Figure 3).

## 4. Discussion

The early diagnosis of gastric cancer is of great importance because effective treatments and a favorable prognosis depend on it. Furthermore, the identification of molecular biomarkers expressed in the initial stages of GC or before the disease appears is an area of major interest. Previous studies have reported that an increase in or diminished expression of miRNAs in serum or whole-blood samples are correlated with the GC stage, nodule invasion [19], and the chance of relapse after a curative resection.

It has been shown that, in addition to being part of the GCSC phenotype described in patients [20], CD54 is important for migration [32]. Our group previously reported a subset of cells lacking CD54/ICAM1 in healthy donors, but present in GC patients, making CD54/ICAM1 a protein of interest in gastric cancer [20]. In this context, we evaluated whether a panel of four miRNAs potentially targeting CD54/ICAM1 could be used as molecular markers for the early diagnosis of GC and the determination of the prognosis in a blood test. CD54 is a glycoprotein and adhesion receptor that is well known for regulating the recruitment of leukocytes to a site of inflammation. It mediates cell adhesion in physiological and pathological conditions such as cancer [32,33].

From the four miRNAs evaluated, hsa-miR-335-5p was the only one detected in the blood plasma of GC patients, but no difference was observed when comparing the relative expression in healthy donors and GC patients. However, in patients who survived longer than 12 months, the downregulation of hsa-miR-335-5p was correlated with a good prognosis. It has been observed that hsa-miR-335-5p acts as a tumor suppressor when it targets Bcl-w in ovarian cancer cell lines [34] or SP1 and Bcl-w in GC, where it suppresses cell invasion but not proliferation [35]. In contrast, hsa-miR-335-5p promotes invasion and metastasis in colorectal cancer [36] and lung adenocarcinoma [37]. Considering that hsa-miR-335 is a molecule that could help in determining a prognosis for patients with GC, it is important to evaluate its role in metastasis and/or migration, as these are some of the causes of relapse in patients with cancer.

In summary, our results demonstrate that hsa-miR-335-5p cannot be used as a biomarker for GC diagnosis but could be used as a biomarker for predicting long-term survival through peripheral blood sample collection. One of the limitations of this work is that only the expression of miRNAs potentially targeting a single molecule, in this case, ICAM1, was investigated. However, GC heterogeneity results in different clinical outcomes, and examining only one aspect or gene target could help to elucidate some of the aspects of this heterogeneity.

## Figures and Tables

**Table 1:** MiRNAs that potentially regulate CD54.

MiRNAs with Binding Sites at ICAM 3′ UTR	Reference	Database/Bibliographic Source	Score
hsa-miR-31	Suarez, 2010 [23]	Bibliography	NA
hsa-miR-17-3p	Suarez, 2010 [23]	Bibliography	NA
hsa-miR-335-5p	Luo, 2018 [22]	Bibliography/miRbase	
hsa-miR-593-5p	Zhang, 2020 [24]	Bibliography	NA
hsa-miR-3975 (MIRT611891)		miRbase	High
hsa-miR-1256 (MIRT005710)	Liu, 2018 [25]	Bibliography/miRbase	High
hsa-miR-335-5p (MIRT018131)	Mahmoudian, 2021 [26]	Bibliography/miRbase	High
hsa-miR-3186-3p (MIRT611892)	Zhang, 2022	Bibliography/miRbase	High

**Table 2:** Clinical characteristics of subjects.

Clinicopathological	Patients			Donors	
Variable	*n* = 38		X^2^	*n* = 41	
	*N*	%		*N*	%
Gender			0.393		
Male	21	44.7		17	41.5
Female	17	55.3		24	58.5
Age group			0.371 ^a^		
Young adult	3	7.9			
Middle-aged adult	21	55.3			
Old adult	14	36.8			
Tumor grade			0.632 ^a^		
Grade I	1	2.6			
Grade II	7	18.4			
Grade III	30	78.9			
Lauren classification			0.337 ^a^		
Intestinal	18	47.4			
Diffuse	18	47.4			
Mixed	1	2.6			
Not applicable	1	2.6			
TNM classification			0.537 ^a^		
I	3	7.9			
IIA	4	10.5			
IIIA	7	18.4			
IIIB	10	26.3			
IIIC	1	2.6			
IV	13	34.2			
T stage			0.237 ^a^		
T1	3	7.9			
T2	0	0			
T3	8	21.1			
T4a	10	26.3			
T4b	17	44.7			
N stage			0.533 ^a^		
N0	11	28.9			
N1	6	15.8			
N2	7	18.4			
N3	14	36.8			
M stage			0.311 ^a^		
M0	23	60.5			
M1	15	39.5			
Borrmann’s classification			0.754 ^a^		
I	3	7.9			
II	1	2.6			
III	21	55.3			
IV	10	26.3			
V	3	7.9			
Metastatic Site			0.078 ^a^		
No metastasis	23	60.5			
Liver	6	15.8			
Pancreas	2	5.3			
Mediastinum	1	2.6			
Lung	3	7.9			
Bone	1	2.6			
Ovary	2	5.3			

^a^ Fisher’s exact test.

## Data Availability

The original contributions presented in the study are included in the article, further inquiries can be directed to the corresponding author.
